# 

**Published:** 1862-01

**Authors:** 


### BOOKS RECEIVED.

*The Placenta, The Organic Nervous System, The Blood, The Oxygen, and the Animal Nervous System, Physiologically Examined. By* John O'Reilly, M. D., *Licentiate and Fellow of the Royal College of Surgeons in Ireland; Resident Fellow of the New York Academy of Medicine; Member of the Medico-Chirurgical College of New York; late Medical Officer to the Oldcastle Workhouse and Fever Hospital, England. New York:* S. S. & W. Wood, 389 *Broadway. London:* John Churchill, *New Burlington Street.*

